# Neutralizing Antibody Formation with OnabotulinumtoxinA (BOTOX^®^) Treatment from Global Registration Studies across Multiple Indications: A Meta-Analysis

**DOI:** 10.3390/toxins15050342

**Published:** 2023-05-17

**Authors:** Joseph Jankovic, Jean Carruthers, Markus Naumann, Patricia Ogilvie, Terry Boodhoo, Mayssa Attar, Swati Gupta, Ritu Singh, John Soliman, Irina Yushmanova, Mitchell F. Brin, Jie Shen

**Affiliations:** 1 Baylor College of Medicine, Houston, TX 77030, USA; 2 Department of Ophthalmology, University of British Columbia, Vancouver, BC V6T 1Z4, Canada; 3 Jean Carruthers Cosmetic Surgery Inc., Vancouver, BC V5Z 4E1, Canada; 4 Department of Neurology and Clinical Neurophysiology, University Hospital, 86156 Augsburg, Germany; 5 Skin Concept, 80333 Munich, Germany; 6 AbbVie, Irvine, CA 92612, USA; 7 Department of Neurology, University of California, Irvine, CA 92697, USA

**Keywords:** aesthetics, blepharospasm, cervical dystonia, hyperhidrosis, migraine disorders, spasticity, bladder, overactive, neurotoxins, type A botulinum toxins

## Abstract

Though the formation of neutralizing antibodies (NAbs) during treatment with botulinum neurotoxin is rare, their presence may nonetheless affect the biological activity of botulinum toxin and negatively impact clinical response. The goal of this updated meta-analysis was to evaluate and characterize the rate of NAb formation using an expanded dataset composed of 33 prospective placebo-controlled and open-label clinical trials with nearly 30,000 longitudinal subject records prior to and following onabotulinumtoxinA treatment in 10 therapeutic and aesthetic indications. Total onabotulinumtoxinA doses per treatment ranged from 10 U to 600 U administered in ≤15 treatment cycles. The NAb formation at baseline and post-treatment was tested and examined for impact on clinical safety and efficacy. Overall, 27 of the 5876 evaluable subjects (0.5%) developed NAbs after onabotulinumtoxinA treatment. At study exit, 16 of the 5876 subjects (0.3%) remained NAb positive. Due to the low incidence of NAb formation, no clear relationship was discernable between positive NAb results and gender, indication, dose level, dosing interval, treatment cycles, or the site of injection. Only five subjects who developed NAbs post-treatment were considered secondary nonresponders. Subjects who developed NAbs revealed no other evidence of immunological reactions or clinical disorders. This comprehensive meta-analysis confirms the low NAb formation rate following onabotulinumtoxinA treatment across multiple indications, and its limited clinical impact on treatment safety and efficacy.

## 1. Introduction

OnabotulinumtoxinA (BOTOX^®^; Allergan, an AbbVie Company, Irvine, CA, USA) is an injectable botulinum neurotoxin (BoNT) that was first approved by the US Food and Drug Administration in 1989 and is effective across multiple therapeutic and aesthetic indications [1]. It is derived from the bacterium *Clostridium botulinum*, and onabotulinumtoxinA inhibits soluble *N*-ethylmaleimide-sensitive factor attachment protein receptor (SNARE)-mediated vesicle fusion in nerve terminals to prevent the release of motor and sensory neurochemicals and proteins [2,3,4,5].

Repeat dosing is an important part of the treatment regimen for many approved indications for onabotulinumtoxinA, with benefits dependent on continued response to treatment. Although most individuals respond to onabotulinumtoxinA over the long term, a small portion of individuals lose clinical response after initially successful treatment [4,6]. In most cases, this is due to inadequate doses and/or suboptimal muscle selection [7]; however, BoNTs are foreign proteins that are injected into the body and, as such, are capable of inducing an immune response. This may lead to the formation of neutralizing antibodies (NAbs) that have the potential to reduce BoNT’s pharmacological activity and could impact clinical performance [6,8,9,10].

Treatment with onabotulinumtoxinA generally results in low rates of NAb formation [1,3]. In a previous meta-analysis, 0.49% of the 2240 subjects were converted from NAb negative at baseline to NAb positive at one or most post-treatment time points across five indications (cervical dystonia, post-stroke spasticity, axillary hyperhidrosis, neurogenic overactive bladder, and glabellar lines) using different routes of administration [4]. Although rare, NAb formation remains a relevant consideration for clinical practice involving onabotulinumtoxinA treatment [6,11] This updated meta-analysis was therefore undertaken to evaluate the frequency of the NAb formation that follows onabotulinumtoxinA treatment based on the clinical study data from nearly 30,000 longitudinal subject records across 10 therapeutic and aesthetic indications. To further explore the risk factors associated with NAb formation, this analysis also examines the relationships between NAb formation and gender, indication (dose route and location), dose level, dosing intervals, and number of treatment cycles. In addition, the impact of NAb formation on clinical safety and efficacy was also evaluated across indications.

## 2. Results

**Figure 1:**
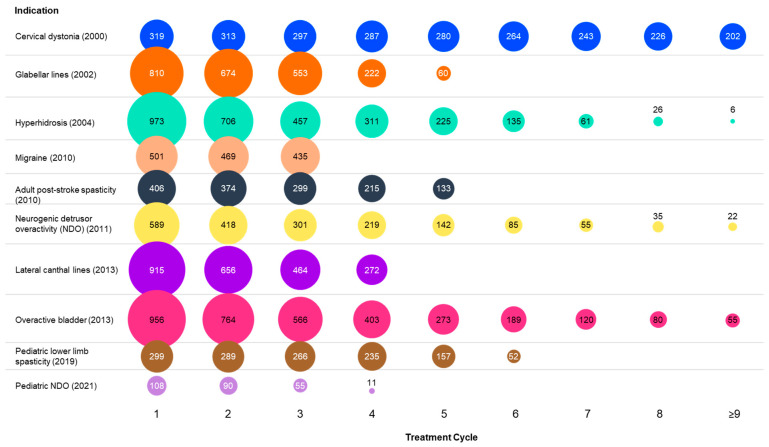
Numbers within or above the circles reflect subject records available for neutralizing antibody evaluation at each treatment cycle (95% of subjects received 1–9 cycles) by approved indication, Indications are ordered by year of onabotulinumtoxinA approval, with approval year in parentheses. Circle sizes are proportional to the number of patient records indicated.

Immunogenicity samples (23,970 in total) were collected from 6146 subjects who had been treated with onabotulinumtoxinA as part of 33 clinical studies across 10 therapeutic and facial aesthetic indications. The study designs and specific inclusion criteria for therapeutic or facial aesthetic indication are presented in Table 1. Prior BoNT treatment was allowed in some trials. Trials of onabotulinumtoxinA that were ongoing were excluded. Of the 33 studies, 31 studies required, per protocol, the collection of blood samples for immunogenicity analysis at study exit. Subjects who only received placebo treatment were not considered any further. Total onabotulinumtoxinA doses per treatment ranged between 10 U (e.g., for glabellar lines) and 600 U (i.e., for adult post-stroke spasticity) (Table 2). Of the 6146 subjects, 5876 had immunogenicity data that allowed the assessment of NAb formation based on prespecified criteria, as is illustrated in Figure 1. Samples were excluded due to insufficient sample volume, positive baseline NAb status, or a lack of post-treatment sample availability. A total of 12 subjects (0.2%) were seropositive for NAb at baseline and were not included in the analysis. Figure 1 shows the number of subject records by injection cycles and by indication based on the data available for NAb evaluation as described. The median discontinuation rate across all studies was 14.7%; therefore, differences in sample sizes by treatment cycle across different indications were driven mainly by study design rather than by subject discontinuations.

### 2.1. Frequency of NAb Formation

Of the subjects with negative or unknown antibody status at baseline, 0% to 1.4% (by individual indication) and 0.5% (for all indications combined) tested NAb positive at any time point post-treatment (Table 3). At the final study exit assessment, only 16/5876 subjects (0.3%) remained NAb positive. No NAbs were detected at study exit for the lateral canthal lines, glabellar lines, migraine, or pediatric neurogenic detrusor overactivity (NDO) indications (Table 3). In addition, although the small number of subjects who were NAb positive precluded statistical analysis, a review of individual subjects found no relationship between NAb formation and baseline subject comorbidities, medical history, or concomitant medications.

**Figure 2:**
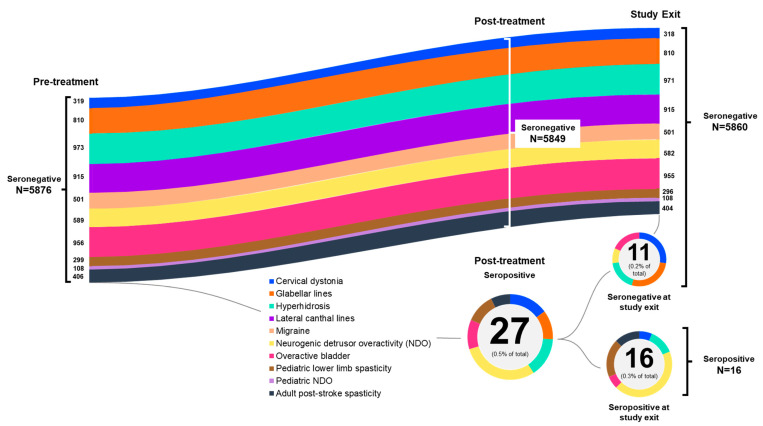
NAb status post-treatment and at study exit of subjects who were seronegative at baseline, by indication. Numbers refer to numbers of subjects. NAb, neutralizing antibody; NDO, neurogenic detrusor overactivity.

As depicted in Figure 2, the numbers of subjects who were seronegative pre-treatment remained seronegative post-treatment and at study exit, or they converted to seropositive.

### 2.2. Effect of Dose Level, Dosing Interval, and Number of Treatment Cycles on Immunogenicity

**Figure 3:**
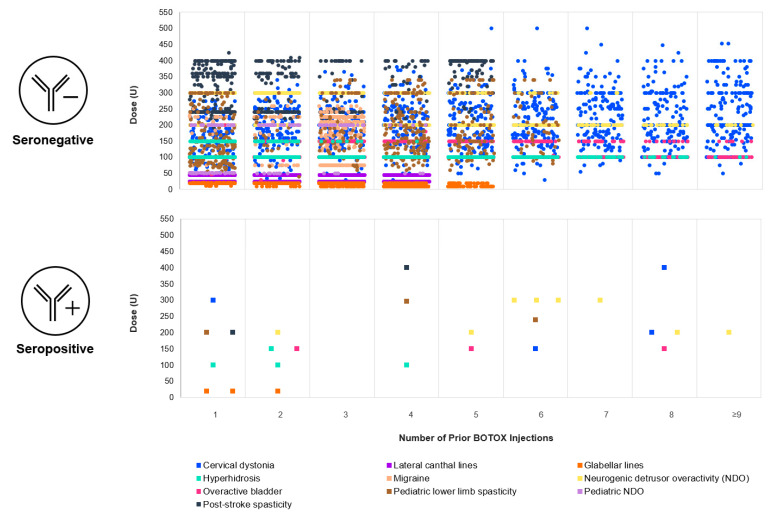
Jitter plot showing subjects who were NAb negative (top) and NAb positive (bottom) by dose and treatment cycle for each approved indication (≈95% of subjects received 1–9 treatment cycles; no subject was positive beyond treatment cycle 9).

Review of the data from the 27 subjects with post-treatment NAbs showed that NAb-positive events were not clearly associated with higher doses or number of treatment cycles (Figure 3).

Table 4 shows the onset incidence of first NAb formation at each treatment cycle of onabotulinumtoxinA across all 10 indications combined. With the exception of cycle 8, all other cycles up to 15 had fewer than 1% of patients first becoming NAb positive in that cycle, and there was no trend of increased incidence as the number of treatments received increased; there were more than 100 evaluable patients through 11 cycles of treatment.

Table 5 shows the mean dosing interval for patients who remained NAb negative throughout the trials compared with patients who became NAb positive in response to onabotulinumtoxinA treatment. The data do not suggest more frequent dosing (i.e., a shorter mean dosing interval) in patients who developed NAb versus those who did not.

### 2.3. Effect of NAb Formation on Efficacy

To assess the impact of NAb formation on clinical efficacy, clinical response was evaluated in the 27 subjects who had NAb formation across seven indications. Based on the timing of NAb formation relative to clinical efficacy assessment and prospectively defined criteria for a responder, the 27 subjects can be divided into four categories: initial responders who lost response after Nab formation (i.e., true secondary nonresponders); initial responders who continued to respond despite NAb formation; initial responders without available efficacy assessments after NAb formation; and nonresponders both prior to and after NAb formation (Table 6). Only five subjects were true secondary nonresponders.

### 2.4. Effect of NAb Formation on Safety

A review of the adverse event profile of the 27 subjects who developed a positive NAb response post-treatment revealed no hypersensitivity reactions or other immune-related adverse events.

## 3. Discussion

This comprehensive and robust meta-analysis confirmed the low frequency of NAb formation following onabotulinumtoxinA treatments in 5876 subjects across 10 different indications for up to 15 cycles at total doses ranging from 10 to 600 U per treatment. The frequency of NAb formation at any post-treatment time point was low (0% to 1.4%), and only 27 of 5876 subjects (0.5%) developed NAbs after treatment across all 10 indications. Due to the low number of subjects with NAbs, no clear relationship can be drawn between positive NAb results and subject gender, age, indication (dose route and location), onabotulinumtoxinA dose level, or number of treatment cycles. Instead, this analysis confirmed that repeat dosing does not predispose subjects to the development of NAbs.

OnabotulinumtoxinA therapy is highly effective across several therapeutic and facial aesthetic conditions, many of which are chronic conditions that require repeated treatments over time. Although rare, the development of NAb-associated immunoresistance is an important consideration during BoNT treatment [1,6,11,44] because NAbs may interfere with BoNT pharmacologic activity and potentially reduce or negate BoNT clinical efficacy [6]. An important finding of the present study is that most subjects continued to respond clinically despite the presence of NAbs. Indeed, only five of the 27 subjects with NAbs who initially responded to onabotulinumtoxinA therapy lost response after NAb formation and could be considered secondary nonresponders; furthermore, three were being treated for cervical dystonia and two for NDO. The majority of those who developed NAbs (14 of 27) were initial responders to onabotulinumtoxinA treatment who maintained treatment response after the development of NAbs. This suggests that NAb development does not necessarily always reduce the efficacy of treatment with onabotulinumtoxinA.

Clinical screening tests, such as the extensor digitorum brevis (EDB) or injections in facial muscles, including the Frontalis Antibody Test (FTAT) and Unilateral Brow Injection (UBI), avoid using animals and are convenient to perform. This lends to their clinical utility in assessing response to BoNT therapy but not for the direct measurement of the presence of Nabs [10,45,46,47,48]. FTAT and UBI tests have been reported to correlate well with clinical response to onabotulinumtoxinA in patients [10,47], whereas the EDB test results correlated well with serum antibody assay results [45,46].

This continuation of an earlier meta-analysis [4] analyzed additional patient populations, including pediatric subjects treated for HH, NDO, and LLS, as well as adult subjects treated for lateral canthal lines and for the prevention of chronic migraine. Similar to adults, the immunogenicity rates were low for pediatric subjects, with no subjects in the HH and NDO groups and only 3/299 in the LLS group developing NAbs. Among the three pediatric subjects who developed NAbs, two continued to respond to onabotulinumtoxinA treatment, whereas one never responded prior to or after NAb formation (Table 6). None of the subjects (n = 501) treated for the prevention of chronic migraine for up to three treatments developed NAbs. OnabotulinumtoxinA has been approved since 2010 for the prevention of chronic migraine. As new treatments emerge for the prevention of migraine, such as monoclonal antibodies that have the potential to stimulate the formation of anti-drug antibodies, onabotulinumtoxinA still remains an established treatment option for the prevention of chronic migraine as it demonstrates low immunogenicity rates after multiple treatment cycles [49,50,51,52,53,54].

The present analysis also added substantially to the aesthetic and adult NDO populations described in the previous meta-analysis [4]. In aesthetics, a total of 1725 subjects were treated for glabellar lines or lateral canthal lines for up to five treatment cycles, and none of the subjects had NAbs at study exit. A total of eight of the 27 subjects in the present meta-analysis with NAbs were treated for NDO, representing 1.4% of the NDO subjects studied. NDO is a common complication of spinal cord injury and multiple sclerosis [55], which is an immune-mediated disorder often treated with immune modulating or suppressing medications [56]. Furthermore, all of the eight subjects with NAbs in the present study population had spinal cord injury. Four of the eight NDO subjects with NAbs continued to respond to treatment (two lost response and two lacked efficacy information), again illustrating the imperfect relationship between NAb formation and clinical response.

The method used in our study to determine NAb status (the MPA) detects a biological response to BoNTs and therefore only gives a positive result for antibodies that interfere with, or neutralize, this response. NAbs are those that develop against selected portions or epitopes of the core BoNT protein, i.e., the 150 kD protein component. In nature, BoNTs have evolved to form a complex containing the 150 kD protein and various non–toxin-associated proteins (NAPs) [2]. OnabotulinumtoxinA contains the 150 kD component in association with NAPs. Antibodies that develop against NAPs do not affect biological activity and are referred to as non-neutralizing [6,57]. The important distinction between the two types of antibodies is that non-neutralizing antibodies are not expected to be biologically or clinically relevant [6]. The MPA is considered a sensitive test for NAbs and is more highly correlated with clinical response than other assays, such as the mouse hemidiaphragm [8,58]. In addition, it has been used to support immunogenicity data generation and label language for all approved indications of onabotulinumtoxinA.

Other studies have evaluated the formation of NAbs among BoNT formulations that differ with regard to the presence of NAPs, which have been suggested to stimulate the immune system and facilitate the development of NAbs [57]. In the present analysis, all clinical studies were conducted with an updated formulation of onabotulinumtoxinA, introduced in 1997, containing substantially reduced levels of neurotoxin protein when compared with the earlier formulation. The incidence of NAb development in clinical trials has been reported to vary from 0% to 1.9% for the reformulated onabotulinumtoxinA, and from 0% to 1.8% for incobotulinumtoxinA, which contains no complex proteins in its formulation [1,44,57,59]. The incidence of NAb development observed in the present analysis of onabotulinumtoxinA, at 0.5%, is consistent with previous studies. However, the comparison of NAb formation rates between different BoNT formulations/products or between studies of the same formulation is challenging for several reasons, including differences in patient populations, indications, study methodologies, assays, and reagents for detecting NAbs, as well as for definitions of clinical response [4,57].

The current study describes low NAb formation rates and doses specific to onabotulinumtoxinA. These cannot be automatically extrapolated to other BoNT products, which have differences in formulation and manufacturing methods. It is important to point out that the low frequency of NAb in our analysis may reflect the number of treatment cycles (up to 15 in cervical dystonia, and with an overall mean treatment number across all of the indications of 3.3). Past studies found that the incidence of NAb increased with the cumulative dose and number/frequency of injection visits [48,60]. However, in the large, controlled dataset analyzed in the present study, very few subjects had NAbs. This was the case regardless of dosing interval, number of treatment cycles, or indication, suggesting that current treatment practices (which have been informed by the aforementioned past studies) contribute to the current low NAb rates. Prospective, longitudinal, comparative clinical studies investigating NAb development across BoNTs have not been conducted.

Injecting the lowest effective dose of onabotulinumtoxinA, with the longest acceptable interval between injections, has been recommended to reduce the potential for antibody development [1,44]. However, subjects may express an interest in receiving toxin injections at shorter intervals for the improved maintenance of neuromodulator activity [6]. It is therefore important to note that our analysis did not show any clear trend between shorter dosing intervals and the development of NAbs to onabotulinumtoxinA. Although some products have been developed with shorter dosing intervals in mind, the literature supports using the lowest possible effective dose and avoiding unnecessary switching between different formulations [8,9]. Good clinical practice supports administering the minimum dose sufficient to provide meaningful efficacy, safety, and duration of effect.

A notable implication of the present study is that poor or no response to onabotulinumtoxinA may be attributed to other factors besides potential immunogenicity and NAbs. This is supported by a study of cervical dystonia subjects in which the most common reasons for an unsatisfactory response to BoNTs were insufficient dosing and suboptimal muscle targeting [7]. Other factors such as improper injection technique and conditions that are challenging to treat, such as preexisting anterocollis or contractures as a result of longstanding abnormal posture, may also contribute [7] and thus merit further study. Differing patient/provider perceptions of treatment benefit, unrealistic treatment expectations, and disease state progression should also be considered.

## 4. Conclusions

Healthcare practitioners and subjects depend on the long-term safety and efficacy of BoNT treatment across a wide array of indications. The carefully controlled and optimized manufacturing process of onabotulinumtoxinA, along with its large body of clinical trial data, support the low incidence of NAbs. This comprehensive and robust meta-analysis is the largest analysis of onabotulinumtoxinA immunogenicity performed to date. The data confirmed that rates of NAb formation are low following onabotulinumtoxinA treatments across multiple therapeutic and aesthetic indications, and that NAb development has limited clinical impact on the safety and efficacy of onabotulinumtoxinA treatment. The findings highlight the importance of re-evaluating the muscles, doses, and even the subject expectations in the event of poor clinical response to onabotulinumtoxinA.

## 5. Materials and Methods

### 5.1. Study Designs and Subjects

The meta-analysis included subjects treated with onabotulinumtoxinA from 33 longitudinal clinical studies that were conducted internally by Allergan (an AbbVie Company) or its business partners, and contained immunogenicity data. The studies adhered to all regulatory guidelines for product licensure. At the time of analysis, all studies were completed with internal sponsor databases locked and with individual subject data and study reports available.

### 5.2. NAb Evaluations

**Figure 4:**
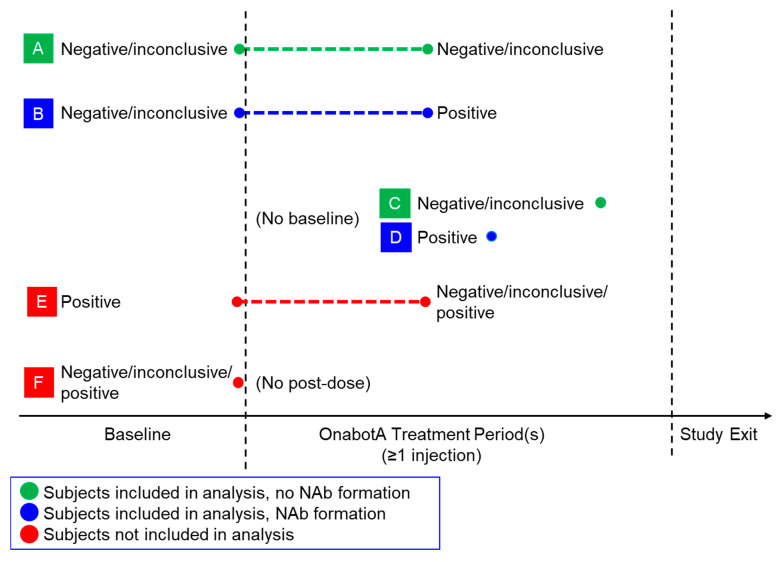
Methodology to determine NAb formation in response to onabotulinumtoxinA treatment based on individual subject scenarios for baseline vs. post-treatment NAb results. Abbreviations—NAb, neutralizing antibody; OnabotA, onabotulinumtoxinA.

The inclusion criteria for subject records and the methodology for evaluating NAb formation are summarized in Figure 4. Subject serum samples were collected and analyzed to determine NAb formation rates and frequency. The in vivo mouse protection assay (MPA) was validated and was used to detect the NAbs in serum samples following treatment, as previously described in [4]. NAbs were detected using either a one-step approach (for studies initiated prior to September 2009) using the MPA alone, or a two-step approach (for studies initiated in September 2009 or later) that included a validated enzyme-linked immunosorbent assay for binding antibody (BAb) that followed the validated MPA. In the two-step approach, serum samples with confirmed positive binding assay results were included for MPA analysis.

For both the one-step or two-step approaches, subjects were evaluated for NAb formation in response to onabotulinumtoxinA treatment based on the following algorithm (with letters in parentheses corresponding to scenarios in Figure 4):

- All samples with NAb results (positive, negative, or inconclusive) were used to determine subjects’ NAb status;
- Subjects with a negative or inconclusive baseline were considered not to have NAb formation if all post-dose serum samples were negative or inconclusive (A), but were considered to have NAb formation if any post-dose sample was positive (B);
- Subjects with no baseline assessment were considered not to have NAb formation if all post-dose samples were negative or inconclusive (C), but were considered to have NAb formation if any post-dose sample was positive (D);
- Subjects were not included in the current analysis if they had positive baselines (E);
- Subjects were not included in the current analysis if they had no post-dose NAb results (F).

Post-treatment follow-up times for sample analysis varied because of differences in the study design, indication, and patient population for onabotulinumtoxinA therapy. Furthermore, baseline serum sample results were not available for all indications (e.g., migraine samples collected at baseline were not analyzed because all post-treatment samples were negative for NAb).

### 5.3. Statistical Analyses

Summary statistics were used for subject demographics, onabotulinumtoxinA doses, and number of injections, based on indication. The numbers and percentages of onabotulinumtoxinA-treated subjects with NAb-positive serum samples at any post-baseline follow-up visit or upon study exit (using the latest result) were calculated for each indication, as well as for all indications combined, with 95% Wald confidence intervals presented. Subjects with NAb-positive samples for the applicable visit(s) were counted in the numerator (B+D from Figure 4), and all evaluable subjects were included in the denominator (A+B+C+D from Figure 4) for these analyses. Incidence rates for first onset of NAb formation were evaluated by treatment cycle, where subjects at risk for a given treatment cycle were those who had not already experienced a positive post-dose NAb in a previous treatment cycle and who had at least one NAb result in the current treatment cycle. To evaluate the effect of dosing intervals on NAb formation, only the subset of subjects with at least two treatment cycles can be evaluated (since subjects with just one treatment cycle do not have any calculable dosing interval). For these subjects, the mean dosing interval prior to the first cycle in which NAb formation was identified was calculated. For reference, the mean dosing interval was also calculated for this subset of subjects who never had NAb formation using all the cycles prior to the cycle with their last NAb result. All calculations were conducted in SAS Version 9.4.

Individual data listings of the subjects who had at least one post-baseline NAb-positive serum result were reviewed to determine clinical responsiveness to onabotulinumtoxinA. Adverse event listings were reviewed for all adverse events, including immune-related events, and the medical history and concomitant medications were reviewed for any comorbidities that could be possibly associated with NAb formation.

## Figures and Tables

**Table 1:** Study characteristics included in the meta-analysis of onabotulinumtoxinA for aesthetic and therapeutic indications.

Study	Inclusion Criteria Related to Prior BoNT Treatment	Design
**Cervical dystonia**		
Brin 2008 [10]	BoNT naive	OL
**Glabellar lines**		
Carruthers 2002 [12]	No requirement	DBPC
Carruthers 2004 [13]	No requirement	OL
Carruthers 2003 [14]	No requirement	DBPC
Kawashima 2009 [15]	BoNT naive	OL
**Primary axillary hyperhidrosis**		
Lowe 2007 [4,16]	No BoNT for this condition previously	DBPC
Glaser 2007 [4,17]	Only in past BoNT study	OL
Glaser 2015 [18]	BoNT naive	OL
Naumann 2001 [19]	No BoNT ≤4 months	DBPC
Naumann 2003 [20]	No requirement	OL
Pariser 2005 [4,21]	BoNT naive	OL
**Migraine**		
Mathew 2005 [22]	BoNT naive	DBPC
Silberstein 2005 [23]	BoNT naive	DBPC
Aurora 2007 [24]	BoNT naive	DBPC
**Adult post-stroke spasticity**		
Brashear 2002 [25]	BoNT naive	DBPC
Gordon 2004 [26]	No requirement	OL
Turkel 2002 [4,27]	No BoNT ≤4 months	DBPC
Elovic 2008 [28]	No BoNT ≤4 months	OL
**Neurogenic detrusor overactivity**		
Schurch 2005 [29]	No BoNT for urologic condition; no BoNT for any indication ≤3 months	DBPC
Cruz 2011, Ginsberg 2012, Ginsberg 2013 [30,31,32], ^a^	No BoNT for urologic condition; no BoNT for any indication ≤3 months	DBPC
Kennelly 2017 [33], ^a^	Only in past BoNT study	OL
Study 082P (data on file)	No BoNT for urologic condition; no BoNT for any indication ≤3 months	DBPC
**Lateral canthal lines**		
Carruthers 2014 [34], ^a^	BoNT naive	DBPC
Moers-Carpi 2015 [35], ^a^	BoNT naive	DBPC
Carruthers 2015 [36], ^a^	Only in past BoNT study	DBPC
**Overactive bladder ^b^**		
Chapple 2013 [37], ^a^	No BoNT for urologic condition; no BoNT for any indication ≤12 weeks	DBPC
Nitti 2013 [38], ^a^	No BoNT for urologic condition; no BoNT for any indication ≤12 weeks	DBPC
Nitti 2016 [39], ^a^	No requirement	OL
Ginsberg 2017 [40], ^a^	No requirement	OL
**Pediatric spasticity**		
Dimitrova 2022 [41], ^a^	No BoNT for any indication ≤6 months	DBPC
Dimitrova 2021 [42], ^a^	No requirement	DBPC
**Pediatric neurogenic detrusor overactivity**		
Austin 2021	No previous/current BoNT for any urologic condition	DBPC
Study 121R (data on file)	No requirement	OL

Indications are ordered by year of onabotulinumtoxinA approval (see Figure 1 for approval years). Abbreviations—BoNT, botulinum toxin; DBPC, double-blind, placebo-controlled; DBPG, double-blind, parallel group; and OL, open label. ^a^ Studies not included in Naumann 2010 [4]. ^b^ Excludes neurogenic causes, which are instead included in neurogenic detrusor overactivity.

**Table 2:** Dosing regimen and subject characteristics by onabotulinumtoxinA indication.

Indication	Subjects, n	Gender (M, F), n (%)	Maximum No. of Treatment Cycles	Mean (SD) No. of Treatment Cycles	Dose Range, U	Mean (SD) Dose, U	Dosing Route
Cervical dystonia	326	98 (30), 228 (70)	15	8.4 (3.22)	20–500	187.3 (76.47)	IM
Glabellar lines	846	100 (12), 746 (88)	5	2.8 (1.15)	10–20	17.2 (4.51)	IM
Hyperhidrosis	1077	439 (41), 638 (59)	14	2.8 (1.97)	100–150	102.8 (11.41)	ID
Migraine	501	69 (14), 432 (86)	3	2.8 (0.53)	75–260	165.2 (57.00)	IM
Adult post-stroke spasticity	449	224 (50), 225 (50)	5	3.3 (1.42)	100–600	307.3 (79.18)	IM
NDO	619	256 (41), 363 (59)	13	3.1 (2.28)	200–300	238.2 (48.59)	IM
Lateral canthal lines	916	109 (12), 807 (88)	4	2.5 (1.19)	24–44	33.8 (10.00)	IM
Overactive bladder ^a^	974	108, (11), 866 (89)	13	3.6 (2.48)	20–200	108.7 (19.26)	IM
Pediatric lower limb spasticity	325	173 (53), 152 (47)	6	4.2 (1.37)	40–340.5	177.7 (76.23)	IM
Pediatric NDO	113	65 (58), 48 (42)	4	2.4 (0.92)	50–200	128.4 (59.85)	IM
**Total**	**6146**	**M: 1641 (27),** **F: 4505 (73)**	**3–15**	**3.3 (2.25)**	**10–600**	**134.8 (94.89)**	**ID, IM**

Indications are ordered by year of onabotulinumtoxinA US Food and Drug Administration approval (see Figure 1 for approval years). Abbreviations—F, female; ID, intradermal; IM, intramuscular; M, male; and NDO, neurogenic detrusor overactivity. ^a^ Excludes neurogenic causes, which are instead included in NDO.

**Table 3:** Frequency of neutralizing antibody detection with onabotulinumtoxinA across multiple indications.

Indication	Subjects, n	Post-Treatment NAb-Positive Subjects, n (%) [95% CI]	Post-Treatment NAb-Positive Subjects by Gender		NAb-Positive Subjects at Study Exit, n (%) [95% CI]
			Male, n (%) [95% CI]	Female, n (%) [95% CI]	
Cervical dystonia	319	4 (1.3) [0, 2.5]	2 (2.1) [0, 4.9]	2 (0.9) [0, 2.1]	1 (0.3) [0, 0.9]
Glabellar lines	810	3 (0.4) [0, 0.8]	0 (0.0)	3 (0.4) [0, 0.9]	0 (0.0)
Hyperhidrosis	973	4 (0.4) [0, 0.8]	1 (0.3) [0, 0.8]	3 (0.5) [0, 1.1]	2 (0.2) [0, 0.5]
Migraine	501	0 (0.0)	0 (0.0)	0 (0.0)	0 (0.0)
Adult poststroke spasticity	406	2 (0.5) [0, 1.2]	1 (0.5) [0, 1.4]	1 (0.5) [0, 1.5]	2 (0.5) [0, 1.2]
NDO	589	8 (1.4) [0.4, 2.3]	8 (3.3) [1.0, 5.5]	0 (0.0)	7 (1.2) [0.3, 2.1]
Lateral canthal lines	915	0 (0.0)	0 (0.0)	0 (0.0)	0 (0.0)
Overactive bladder	956	3 (0.3) [0, 0.7]	0 (0.0)	3 (0.4) [0, 0.7]	1 (0.1) [0, 0.3]
Pediatric lower limb spasticity	299	3 (1.0) [0, 2.1]	2 (1.3) [0, 3.1]	1 (0.7) [0, 2.1]	3 (1.0) [0, 2.1]
Pediatric NDO	108	0 (0.0)	0 (0.0)	0 (0.0)	0 (0.0)
**Total**	**5876**	**27 (0.5% of total)** **[0.3, 0.6]**	**14 (0.9)** **[0.4, 1.4]**	**13 (0.3)** **[0.1, 0.5]**	**16 (0.3% of total)** **[0.1, 0.4]**

Indications are ordered by year of onabotulinumtoxinA approval (see Figure 1 for approval years). Abbreviations—F, female; M, male; NAb, neutralizing antibody; and NDO, neurogenic detrusor overactivity.

**Table 4:** Positive NAb onset incidence per treatment cycle for onabotulinumtoxinA across all 10 indications.

Treatment Cycle	Number of Patients with Positive NAb for the First Time	Total Number of Patients Receiving OnabotulinumtoxinA ^a^	Incidence of Positive NAb Onset (%) [95% CI]
1	6	4278	0.1 [0, 0.3]
2	5	2959	0.2 [0, 0.3]
3	0	2584	0
4	3	1624	0.2 [0, 0.4]
5	2	1001	0.2 [0, 0.5]
6	5	610	0.8 [0.1, 1.5]
7	1	422	0.2 [0, 0.7]
8	4	334	1.2 [0, 2.4]
9	1	259	0.4 [0, 1.1]
10	0	176	0
11	0	116	0
12	0	49	0
13	0	17	0
14	0	6	0
15	0	1	0

NAb, neutralizing antibody. ^a^ Number of patients who had evaluable immunogenicity data and had not yet developed positive NAbs up to that treatment cycle. Not all patients had NAb results for every treatment cycle.

**Table 5:** Comparison of average dosing interval (days) in subjects with and without NAb formation across indications.

NAb Formation	Lower Quartile	Median	Upper Quartile
No (n = 4449)	96.5	119	196
Yes (n = 21) ^a^	101	134	186

NAb, neutralizing antibody. ^a^ Note that six of the 27 subjects who were positive for NAb at anytime post-dose had their first NAb formation following first treatments; therefore, they were not eligible to be included in this analysis as there was no prior dosing interval to calculate.

**Table 6:** Categorization of clinical response ^a^ in subjects who had Nab formation.

Indications	Initial Responders			Nonresponders Both Prior to and after Nab Formation [D] ^c^	Total
	Lost Response after Nab Formation (Secondary Nonresponders) [A]	Continued to Respond after Nab Formation [B]	No Efficacy Results Available after Nab Formation [C]		
Cervical dystonia	3	1	0	0	4
Glabellar lines	0	3 ^b^	0	0	3
Hyperhidrosis	0	3	1	0	4
Adult post-stroke spasticity	0	1	0	1	2
Neurogenic detrusor overactivity	2	4	2	0	8
Overactive bladder	0	1	0	2	3
Pediatric lower limb spasticity	0	2	0	1	3
**Total**	**5**	**15**	**3**	**4**	**27**

Indications are ordered by year of onabotulinumtoxinA approval (see Figure 1 for approval years). Nab, neutralizing antibody. ^a^ Clinical response defined as in [4]. Cervical dystonia: investigator judgment of clinical response at time of re-treatment (yes/no); Glabellar lines: on the four-point physician assessment score, improvement of 1 or 2 points from a baseline severity score of moderate (2) or severe (3); Hyperhidrosis: ≥50% reduction from session baseline sweating by gravimetric measurement or a score of 1 or 2 on the Hyperhidrosis Disease Severity Scale at weeks 4 and 8 post-injection and ≥50% reduction from session baseline sweating, as measured by gravimetric measurement; Adult post-stroke spasticity: ≥1-point decrease from baseline for any treated muscle group on the Ashworth Scale; Neurogenic detrusor overactivity: ≥50% reduction from session baseline in daily urinary incontinence episodes; Overactive bladder: ≥50% reduction from session baseline in daily urinary incontinence episodes; Pediatric lower limb spasticity: ≥1-point decrease from baseline for ankle score with knee extended on the Modified Ashworth Scale [43]; ^b^ This includes one subject who was a nonresponder prior to NAb formation but became a responder after NAb formation. ^c^ This category also includes subjects who were nonresponders prior to NAb formation and did not have any clinical assessment after NAb formation.

## Data Availability

AbbVie is committed to responsible data sharing regarding the clinical trials we sponsor. This includes access to anonymized, individual, and trial-level data (analysis data sets), as well as for other information (e.g., protocols, clinical study reports, or analysis plans) as long as the trials are not part of an ongoing or planned regulatory submission. This includes requests for clinical trial data for unlicensed products and indications. These clinical trial data can be requested by any qualified researchers who engage in rigorous, independent, scientific research, and will be provided following review and approval of a research proposal, a Statistical Analysis Plan (SAP), and the execution of a Data Sharing Agreement (DSA). Data requests can be submitted at any time after approval in the US and Europe and after acceptance of this manuscript for publication. The data will be accessible for 12 months, with possible extensions considered. For more information on the process or to submit a request, visit the following link: https://www.abbvieclinicaltrials.com/hcp/data-sharing.
